# Quantitative comparison of dynamic physiological feeding profiles for recombinant protein production with *Pichia* *pastoris*

**DOI:** 10.1007/s00449-013-1087-z

**Published:** 2013-11-10

**Authors:** Oliver Spadiut, Denes Zalai, Christian Dietzsch, Christoph Herwig

**Affiliations:** Institute of Chemical Engineering, Research Area Biochemical Engineering, Vienna University of Technology, Gumpendorfer Strasse 1a, 1060 Vienna, Austria

**Keywords:** *Pichia* *pastoris*, Recombinant protein, Fed-batch cultivation, Stress, Dynamic feeding profile

## Abstract

*Pichia* *pastoris* is widely used for the production of recombinant proteins in industrial biotechnology. In general, industrial production processes describe fed-batch processes based on the specific growth rate. Recently, we introduced the specific substrate uptake rate (*q*_s_) as a novel parameter to design fed-batch strategies for *P.* *pastoris*. We showed that a dynamic feeding strategy where the feed was adjusted in steps to the maximum specific substrate uptake rate was superior to more traditional strategies in terms of specific productivity. In the present study, we compare three different dynamic feeding strategies based on *q*_s_ for a recombinant *P.* *pastoris* strain with respect to cell physiology, methanol accumulation, productivity and product quality. By comparing (A) a feeding profile at constant high *q*_s_, (B) a periodically adjusted feeding profile for a stepwise *q*_s_ ramp, and (C) a feeding profile at linear increasing *q*_s_, we evaluated potential effects of the mode of feeding. Although a dynamic feeding strategy with stepwise increases of *q*_s_ to *q*_s max_ resulted in the highest specific productivity, a feeding profile where the feeding rate was stepwise increased to a constant high *q*_s_ value was superior in terms of the amount of active enzyme produced and in the amount of accumulated methanol. Furthermore, this feeding strategy could be run automatically by integrating an online calculator tool, thus rendering manual interventions by the operator unnecessary.

## Background

The eukaryotic, methylotrophic yeast *Pichia* *pastoris* is an important host organism for the production of recombinant proteins. It can grow on inexpensive media to high cell densities, several tools for genetic manipulation are available, and it is capable of performing post-translational modifications and can use methanol as substrate as well as inducer for recombinant protein production. The gene encoding the recombinant target protein can be inserted downstream of two promoters P*aox*1 or P*aox*2 resulting in the two phenotypes, methanol utilization plus (Mut^+^) and methanol utilization slow (Mut^S^) [1–3]. Although the findings in literature with respect to the preferred phenotype are controversial (e.g. [4–7]), we have shown the Mut^S^ phenotype of the *P.* *pastoris* strain KM71H to be superior to the Mut^+^ phenotype for the recombinant production of the enzyme horseradish peroxidase (HRP) [8].

Industrial production processes with *P.* *pastoris* mainly describe fed-batch processes, where the substrate methanol is constantly provided at a defined rate. A lot of different strategies based on the Invitrogen protocol (http://tools.invitrogen.com) have been developed for *P.* *pastoris* to optimize the volumetric productivity, a parameter of great relevance in industry. Most of these strategies employ the specific growth rate (*μ*) as a key parameter to develop either a feeding profile, where *μ* is controlled [3, 9–11], or a feed forward regime, where *μ* is not controlled (e.g. [9, 12, 13]). In a previous study, we have successfully used the specific substrate uptake rate (*q*_s_) as an alternative physiological parameter to develop and test different feeding strategies for *P.* *pastoris* [14]. We found that a dynamic feeding strategy, where the cells on the one hand had time to adapt to methanol, but were then challenged repeatedly by stepwise increasing of *q*_s_ to *q*_s max_, resulted in a higher specific productivity (*q*_p_) compared to more traditional feeding profiles [14]. In subsequent studies, we have shown the broad applicability of this method to different *P.* *pastoris* phenotypes expressing different target proteins in a single as well as in a mixed feed environment with glycerol and methanol [15, 16], underlining the great potential of this dynamic approach for bioprocess development and optimization.

In the present study, we compared different types of dynamic, physiological feeding profiles based on *q*_s_ for a recombinant *P.* *pastoris* strain and analysed cell physiology, methanol accumulation, productivity and product quality. We wanted to see if the mode of dynamic feeding can influence the productivity and to give recommendations for bioprocess design using dynamic processing modes with respect to product quality and quantity.

## Materials and methods

The experiments conducted in this study were performed according to our previous studies [14, 15] and are thus only described briefly here.

### Microorganism

A *P.* *pastoris* KM71H Mut^S^ strain carrying the recombinant gene for the HRP isoenzyme C1A was provided by Prof. Anton Glieder (Graz University of Technology, Austria). Efficient secretion of HRP into the cultivation broth was facilitated by fusion of the prepro signal sequence of the *Saccharomyces cerevisiae* mating factor alpha to the N-terminus of HRP.

### Culture media

Precultures were performed in complex yeast nitrogen base medium (YNBM), whereas fed-batch cultivations were done in defined twofold concentrated basal salt medium (BSM; [17]). The glycerol feed was prepared with glycerol (250 g L^−1^), trace element solution PTM1 (12 mL L^−1^) and antifoam Struktol J650 (0.3 mL L^−1^). The methanol feed was composed of methanol (300 g L^−1^), PTM1 (4 mL L^−1^) and Struktol J650 (0.3 mL L^−1^). Trace element solution (PTM1), per litre: CuSO_4_·5H_2_O, 6.0 g; NaI 0.08 g; MnSO_4_·H_2_O, 3.0 g; Na_2_MoO_4_·2H_2_O, 0.2 g; H_3_BO_3_, 0.02 g; CoCl_2_, 0.5 g; ZnCl_2_, 20.0 g; FeSO_4_·7H_2_O, 65.0 g; biotin, 0.2 g, H_2_SO_4_, 5 mL. The induction period for HRP expression was carried out in the presence of the haeme precursor δ-Aminolevulinic acid (δ-ALA; 1 mM). The concentration of the base NH_4_OH was determined by titration with 0.25 M potassium hydrogen phthalate (KHP).

### Experimental procedure

#### Preculture

Frozen stocks (−80 °C) were cultivated in 100 mL of YNBM in 1,000 mL shake flasks at 30 °C and 230 rpm for maximal 24 h. Then, the preculture was transferred aseptically to the respective culture vessel. The inoculation volume was 10 % of the final starting volume.

#### Fed-batch cultivations

Fed-batch cultivations were carried out in a 5-L working volume glass bioreactor (Infors, Switzerland) in twofold concentrated BSM at 28 °C and 1,495 rpm. The culture was aerated with at least 2 vvm dried air to keep dissolved oxygen levels above 30 %. In case, air flow was limited, pure oxygen was added. Off-gas was measured using an infrared cell for CO_2_ and a zirconium dioxide sensor for O_2_ concentration (DasGip, Germany). Process parameters were recorded and logged in the Lucullus process information management system (PIMS; Lucullus, SecureCell, Switzerland). The fed-batch feed was measured and controlled using a gravimetrically based PID flow controller. Methanol feed was added via a submerged tube. The fed-batch experiments conducted were as follows: after a batch phase on 40 g L^−1^ glycerol, an exponential fed-batch phase was implemented with a controlled specific growth rate of *μ* = 0.15 h^−1^. The exponential glycerol feed was terminated as the volume in the bioreactor reached 2.5 L. Based on OD_600_ measurements, the biomass in the reactor after the batch and the fed-batch phase was determined. Then, the induction phase was started with a methanol feed corresponding to a *q*_s_ setpoint of 0.5 Cmmol g^−1^ h^−1^ to ensure the adaptation of the culture to methanol. After the CO_2_ signal in the off-gas had passed its maximum, the cells were regarded as adapted to methanol. A sample was taken and total biomass was calculated based on OD_600_ and volume measurements before the *q*_s_-based feeding regime was initiated. At several time points during fed-batch cultivations, samples were taken and analysed for accumulated methanol, biomass concentration (dry cell weight and optical density) and enzymatic activity. Accurate *q*_s real_ values were calculated offline based on the online measurements of feed balances and reactor volume and the determined dry cell weight (DCW).

#### Feeding profiles

**Scheme 1:**
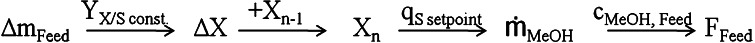
Steps of the automatic feeding strategy applied in this study

All the feeding profiles in this study described feed forward regimes with either an exponential or an accelerated exponential function. To calculate the required exponent for the calculation tool, both a constant biomass composition and a constant biomass yield of *Y*_*X*/*S*_ = 0.38 Cmol Cmol^−1^ were assumed [14]. In order to allow automated, dynamic feeding without the need of extensive interaction of the operator, as we described recently [14, 15], the Lucullus built-in online calculation tool was used. This tool constantly calculated the biomass formed in the bioreactor (Δ*X*) by assuming a constant biomass yield and using the signals delivered from the feed balance. These values were then automatically added to the biomass values determined before (*X*_*n*−1_) to give the apparent actual biomass in the bioreactor. Based thereon and the predefined *q*_s setpoint_, the calculation tool determined the required amount of methanol (m_MeOH_), which had to be fed to maintain a certain *q*_s_ value. Finally, these values in combination with the knowledge of the methanol concentration in the feed, which we had determined by HPLC before, were used to automatically calculate the feeding rate. This automatic feeding strategy is schematically illustrated in Scheme 1.

The underlying equations to calculate the different important process parameters are shown in the following equations:

1 $$S_{\text{in}} = \frac{{\Updelta m_{\text{feed,in}} }}{{\rho_{\text{feed}} }} \cdot c_{{S,{\text{feed}}}}$$

2 $$\Updelta X = S_{\text{in}} \cdot Y_{X/S}$$

3 $$X_{n} = X_{n - 1} + \Updelta X$$

4 $$\dot{S} = \left( {\frac{{q_{{S,{\text{setpoint}}}} }}{1000} \cdot M_{S} } \right) \cdot X_{n}$$

5 $$F_{{{\text{feed}},{\text{setpoint}}}} = \frac{{\dot{S}}}{{c_{{S,{\text{feed}}}} }} \cdot \rho_{\text{feed}}$$

**Fig. 1:**
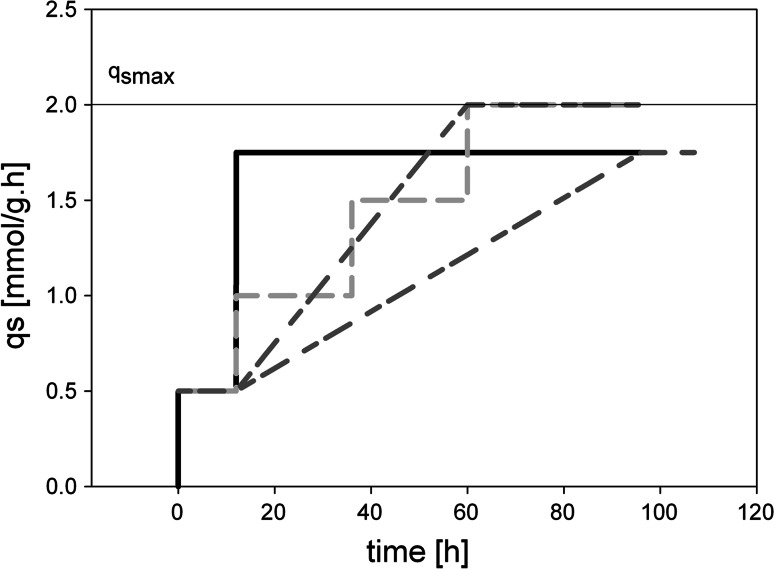
Scheme of different dynamic feeding strategies based on *q*_s_ for a *P.* *pastoris* Mut^S^ strain expressing the recombinant enzyme HRP C1A. After a batch and fed-batch phase on glycerol and an adaptation phase on methanol at a feeding rate corresponding to a *q*_s_ of 0.5 mmol g^−1^ h^−1^, the feeding strategies diverged. *Black solid line* Feeding strategy A: feed was automatically adjusted to correspond to a high *q*_s_ of 1.75 mmol g^−1^ h^−1^; *light grey dashed line* feeding strategy B: the feed was stepwise increased to *q*_s max_ with step sizes of 0.5 mmol g^−1^ h^−1^ every 24 h; *dark grey dashed lines* feeding strategy C: the feed was linearly increased to a *q*_s_ of 1.75 mmol g^−1^ h^−1^ or to *q*_s max_ (different slopes were tested). All the strategies tested described feed forward regimes

We tested three different dynamic feeding profiles based on *q*_s_ (schematically shown in Fig. 1) and compared them in terms of cell physiology, methanol accumulation, productivity and product quality, which we defined as the amount of active enzyme of the total extracellular protein content:

##### Feeding profile at constant high *q*_s_

The feeding ramp was automatically started with a setpoint of *q*_s_ = 0.5 mmol g^−1^ h^−1^ during the adaptation phase, then increased up to 1.75 mmol g^−1^ h^−1^ and automatically kept constant at this value until the end of cultivation. This strategy describes an exponential feeding profile with the function *F* = *F*_0_·e^*k*·*t*^ [*F*, feeding rate (g L^−1^); *F*_0_, initial feeding rate (g L^−1^); *e*, Euler’s number; *k*, exponential coefficient; *t*, time (h)].

##### Periodically adjusted feeding profile for a stepwise *q*_s_ ramp

In this experiment, feeding rates were manually adjusted corresponding to the current total biomass content and the defined *q*_s_ setpoint. After the adaptation period, the *q*_s_ setpoint was set to 1.0 mmol g^−1^ h^−1^. This setpoint was maintained for 24 h and then increased in steps of 0.5–2.0 mmol g^−1^ h^−1^ (Fig. 1). Six samples were collected during each step and the feeding rates were adjusted manually based on measured OD_600_ values [14]. This strategy describes an exponential feeding profile with stepwise increases of the exponent e over time.

##### Feeding profile at linear increasing *q*_s_

After the adaptation phase, the feed was automatically increased linearly either to a *q*_s max_ = 2.0 mmol g^−1^ h^−1^ within 48 h or to a *q*_s_ = 1.75 mmol g^−1^ h^−1^ within 72 h (Fig. 1). This strategy describes an accelerated exponential feeding profile with the function *F* = *F*_0_·e^(*a*+*k*·*t*)·*t*^ [*F*, feeding rate (g h^−1^); *F*_0_, initial feeding rate (g h^−1^); *e*, Euler’s number; *a*, acceleration coefficient; *k*, exponential coefficient; *t*, time (h)].

The different fed-batch experiments and the respective feeding profiles are summarized in Table 1.

**Table 1:** Fed-batch strategies based on *q*_s_ tested in this study

Experiment	Fed-batch strategy	Feeding profile	Feed adjustment
FB1	Feeding profile at constant high *q*_s_	Exponential	Automatically
FB2	Periodically adjusted feeding profile for a stepwise *q*_s_ ramp	Exponential with incremental exponents	Manually adjusted steps of *q*_s_
FB3	Feeding profile at linear increasing *q*_s_	Accelerostat	Automatically
FB4		Accelerostat	Automatically

### Analysis of growth and expression parameters

Dry cell weight (DCW), OD_600_, substrate concentration as well as the catalytic activity of HRP and protein content were determined as described before [14]. Concentrations of methanol and glycerol were determined in cell-free samples by HPLC (Agilent Technologies, USA) equipped with a Supelcoguard column, a Supelcogel C-610H ion-exclusion column (Sigma-Aldrich, USA) and a refractive index detector (Agilent Technologies, USA). The mobile phase was 0.1 % H_3_PO_4_ with a constant flow rate of 0.5 mL min^−1^ and the system was run isocratic. Calibration was done by measuring standard points in the range of 0.1–20 g L^−1^ for each substrate.

### Specific rate calculation

During different cultivation periods, representing defined *q*_s_ setpoints, samples were taken and analysed for OD_600_, DCW, methanol accumulation and product concentration. Specific rates were calculated using DCW and the amount of consumed methanol and produced HRP, respectively. For yield calculations, we used a molar composition of the biomass of 25.74 g Cmol^−1^ based on elemental analysis.

### Data analysis

Measurements of biomass, product and substrate concentration were executed in duplicates. Along the observed standard deviation for these measurements, the error was propagated to the specific rates *q*_s_ and *q*_p_ as well as to the yield coefficients. The error of determination of the specific rates and yields was therefore set to 10 and 5 %, respectively. Carbon and degree of reduction balances were calculated for each sampling period. The acceptance criterion was set to be ±10 % of closing balances.

## Results and discussion

We performed several fed-batch cultivations describing different dynamic feeding profiles based on the physiological parameter *q*_s_ to optimize the feeding strategy for a recombinant *P.* *pastoris* strain. In previous studies, we have shown the validity of the physiological parameter *q*_s_ to develop and conduct bioprocesses with the yeast *P.* *pastoris* as well as several advantages of performing dynamic experiments instead of employing more traditional strategies [8, 14–16, 18]. In the present study, we analysed the effects of different dynamic feed forward regimes on cell physiology, methanol accumulation, productivity and product quality. In all the experiments conducted in this study, an exponential fed-batch cultivation with glycerol yielded DCW concentrations of up to 60 g L^−1^ in a volume of 2.5 L. Following that, we tested three different dynamic feed forward strategies (i.e. high constant *q*_s_ setpoint, stepwise increasing of the *q*_s_ setpoint to *q*_s max_ and linear increasing of the *q*_s_ setpoint to either *q*_s_ of 1.75 mmol g^−1^ h^−1^ or *q*_s max_; Fig. 1; Table 1) to find a strategy which gives high productivities and high product quality and concomitantly prevents extensive methanol accumulation.

### Cell physiology and methanol accumulation

#### Feeding profile at constant high *q*_s_ (exponential feeding profile)

Our first strategy representing a feed forward regime with the function *F* = *F*_0_·e^*k*·*t*^ was similar to the one we described recently [14]. However, in the present study, we improved this strategy by applying an automated feeding regime using the Lucullus built-in online calculator tool (Scheme 1). For the calculation of the exponent, we assumed a constant biomass yield of 0.38 Cmol Cmol^−1^ [14] and a constant biomass composition over the whole induction time. By recording the consumed methanol feed of a known concentration, the online tool calculated the required feeding setpoint (Scheme 1).

**Fig. 2:**
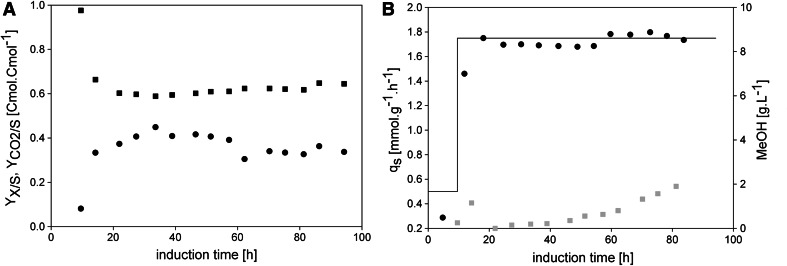
Cell physiology and methanol accumulation in FB1. **a** Biomass and carbon dioxide yields. *Black dots*, *Y*_*X/S*_; *black squares*, $Y_{{{\text{CO}}_{2} /{\text{S}}}}$. **b** Specific substrate uptake rates and methanol concentration in the cell-free cultivation broth. *Black* *solid line*, *q*_s_ setpoint; *black dots*, *q*_s real_; *grey squares*, methanol determined in the cell-free cultivation broth in g L^−1^

In Fig. 2a, the biomass yield (*Y*_*X*/*S*_) and the carbon dioxide yield ($Y_{{{\text{CO}}_{2} /{\text{S}}}}$) are shown. The biomass yield showed an average value of 0.38 Cmol Cmol^−1^, which is in good agreement with the value we determined in our previous study [14], and stayed quite constant over the first 40 h of induction. However, after around 40 h *Y*_*X*/*S*_ started to decrease (Fig. 2a), whereas $Y_{{{\text{CO}}_{2} /{\text{S}}}}$ slightly increased. In Fig. 2b, the *q*_s_ setpoint is compared to the *q*_s real_ values determined throughout induction showing a high correlation. However, although a *q*_s_ value below the *q*_s max_ of 2.0 mmol g^−1^ h^−1^ [14] was adjusted, methanol accumulated in the fermentation broth over time. The starting point of methanol accumulation actually happened at around 40 h of induction concomitantly with the decrease of *Y*_*X/S*_ (compare Fig. 2a and b). Apparently, the assumption of a constant biomass yield over time did not hold true—over cultivation time, the physiology of the cells changed and *Y*_*X/S*_ decreased, which is why methanol in the cultivation broth accumulated.

#### Periodically adjusted feeding profile for a stepwise *q*_s_ ramp (exponential feeding profile with stepwise increases of the exponent over time)

**Fig. 3:**
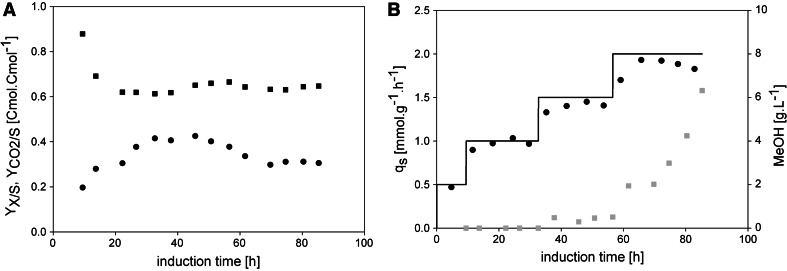
Cell physiology and methanol accumulation in FB2. **a** Biomass and carbon dioxide yields. *Black dots*, *Y*_*X/S*_; *black squares*, $Y_{{{\text{CO}}_{2} /{\text{S}}}}$. **b** Specific substrate uptake rates and methanol concentration in the cell-free cultivation broth. *Black* *solid line*, *q*_s_ setpoint; *black dots*, *q*_s real_; *grey squares*, methanol determined in the cell-free cultivation broth in g L^−1^

To compare the feeding profile at a constant *q*_s_ of 1.75 mmol g^−1^ h^−1^ with a more stressful, dynamic feeding strategy, we conducted FB2, where we increased the feeding rate in steps to *q*_s max_ according to our previous study [14]. The average *Y*_*X/S*_ during the whole induction phase was determined with 0.36 Cmol Cmol^−1^ and was again close to the predetermined biomass yield of 0.38 Cmol Cmol^−1^. However, after around 40 h of induction, the biomass yield started to decrease (Fig. 3a) [14], which was again accompanied by a slight increase of $Y_{{{\text{CO}}_{2} /{\text{S}}}}$ and concomitant methanol accumulation (Fig. 3b). Figure 3b shows the *q*_s_ setpoint and the *q*_s__real_ values, which we determined offline. Again, these values showed a very high correlation. With respect to methanol accumulation, this more stressful, dynamic method resulted in the accumulation of nearly 3 times more methanol than the feeding profile in FB1, especially in the latter phase of induction (Fig. 3b). After around 80 h of induction, more than 6.0 g L^−1^ methanol accumulated. In contrast to FB1, in this experiment we adjusted the feeding rate to reach the maximum specific substrate uptake rate *q*_s max_ of 2.0 mmol g^−1^ h^−1^. To reach *q*_s max_, the amount of fed methanol had to be increased, since these two variables are linked by a Monod-type relationship. In fact, when we set the feeding rate to a value corresponding to *q*_s max_ and thus increased the amount of fed methanol, we observed sudden extensive methanol accumulation (at around 60 h of induction; Fig. 3b), which underlines the importance of this strain-specific parameter—the closer this critical value is approached, the higher the risk of methanol accumulation.

#### Feeding profile at linear increasing *q*_s_ (accelerated exponential feeding profile)

**Fig. 4:**
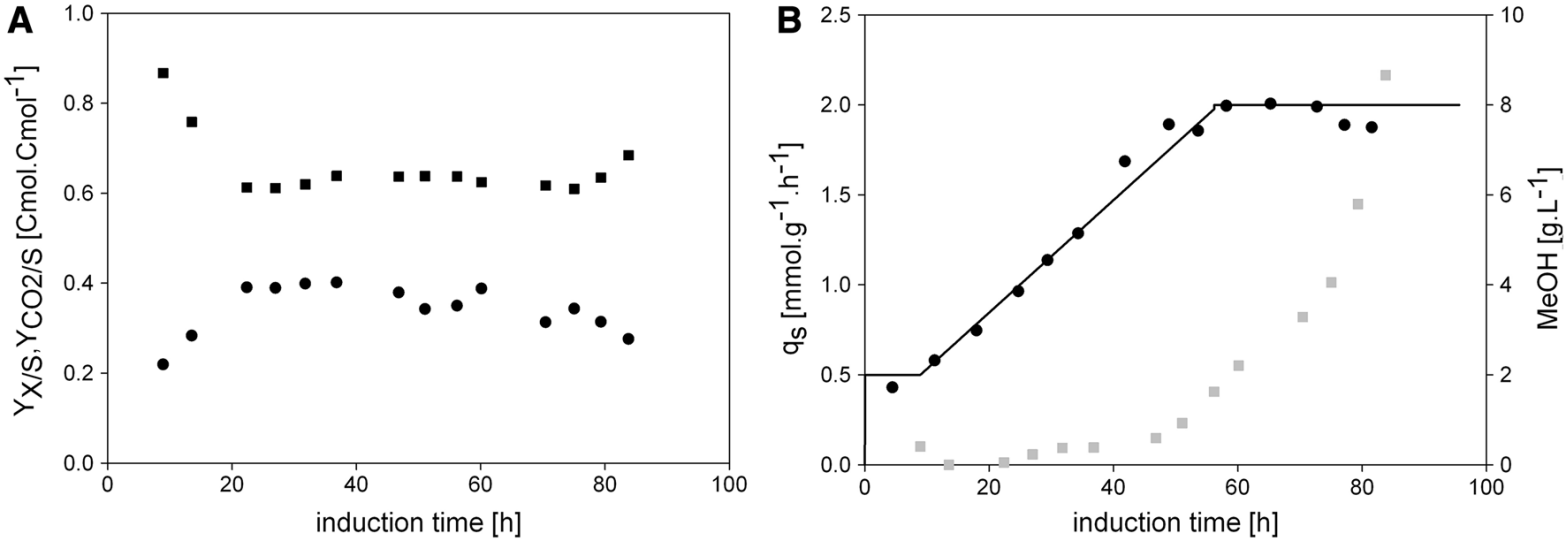
Cell physiology and methanol accumulation in FB3. **a** Biomass and carbon dioxide yields. *Black dots*, *Y*_*X/S*_; *black squares*, $Y_{{{\text{CO}}_{2} /{\text{S}}}}$. **b** Specific substrate uptake rates and methanol concentration in the cell-free cultivation broth. *Black* *solid line*, *q*_s_ setpoint; *black dots*, *q*_s real_; *grey squares*, methanol determined in the cell-free cultivation broth in g L^−1^

To potentially reduce the extensive methanol accumulation observed in FB2, which is especially relevant for large scales, we tested a more gentle feeding approach by employing a linear ramp of *q*_s_ (i.e. an accelerated exponential feeding profile). In FB3, we linearly increased the *q*_s_ setpoint after the adaptation phase from *q*_s_ = 0.5 mmol g^−1^ h^−1^ to *q*_s max_ = 2.0 mmol g^−1^ h^−1^ within 48 h (Fig. 4b) and analysed strain physiology and methanol accumulation. The average *Y*_*X/S*_ in this experiment was determined with 0.35 Cmol Cmol^−1^ and we again observed a decrease of *Y*_*X/S*_ and a slight increase of $Y_{{{\text{CO}}_{2} /{\text{S}}}}$ as well as methanol accumulation after around 40 h of induction. The offline determined *q*_s real_ values were very close to the set ones (Fig. 4b), showing the applicability of our approach of using an online calculator tool for automated feeding. However, methanol accumulation in this experiment was extensive. The closer the feeding strategy approached *q*_s max_, more methanol was fed and the timely decrease in *Y*_*X/S*_ resulted in overfeeding of the cells. However, as long as the methanol concentration stays at levels below *q*_s max_, no extensive methanol accumulation should be observed. The cells simply take up the additional methanol and grow at *q*_s_ higher than the envisioned *q*_s_ setpoint. However, in the FB3 presented here, the feed was adjusted to the *q*_s max_ of the cells, which is why they did not have the capacity to deal with the additional methanol, resulting in excessive accumulation. Summarizing, due to the fact that the cells were fed at their maximum substrate uptake capacity and due to their decreasing *Y*_*X/S*_, methanol accumulated extensively. At the end of cultivation close to 9 g L^−1^ methanol was accumulated. Thus, this strategy bears the risk of harming the cells and/or the product.

**Fig. 5:**
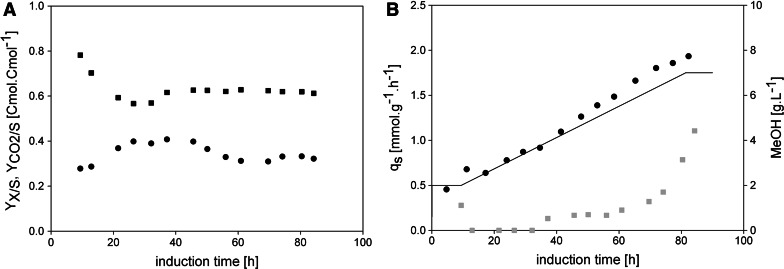
Cell physiology and methanol accumulation in FB4. **a** Biomass and carbon dioxide yields. *Black dots*, *Y*_*X/S*_; *black squares*, $Y_{{{\text{CO}}_{2} /{\text{S}}}}$. **b** Specific substrate uptake rates and methanol concentration in the cell-free cultivation broth. *Black* *solid line*, *q*_s_ setpoint; *black dots*, *q*_s real_; *grey squares*, methanol determined in the cell-free cultivation broth in g L^−1^

To reduce methanol accumulation, we performed FB4, where we applied a smoother ramp linearly increasing the *q*_s_ from 0.5 mmol g^−1^ h^−1^ to only 1.75 mmol g^−1^ h^−1^ within 72 h. In this experiment, the average *Y*_*X/S*_ was determined with 0.36 Cmol Cmol^−1^, which was in the range we determined before [14]. However, after 40 h of induction, we observed a decrease in *Y*_*X/S*_, a slight increase in $Y_{{{\text{CO}}_{2} /{\text{S}}}}$ and methanol accumulation once again (Fig. 5a). From that time point on also the *q*_s set_ and *q*_s real_ values deviated. The real *q*_s_ values exceeded the set *q*_s_ value of 1.75 mmol g^−1^ h^−1^ (Fig. 5b). This is in good agreement with our observations made in FB3: the biomass yield of the cells decreases over time which is why the automatic feeding system overfeeds the cells with methanol. However, in contrast to FB3, in FB4 the *q*_s_ setpoint value was selected below *q*_s max_, which is why the cells had the possibility to cope with the additional methanol by simply taking it up. Thus, the real *q*_s_ values determined offline were significantly higher than the set ones. However, although we were able to reduce methanol accumulation by applying a smoother feeding profile, which ended at *q*_s_ = 1.75 mmol g^−1^ h^−1^ instead of *q*_s max_ = 2.0 mmol g^−1^ h^−1^, still 4.5 g L^−1^ methanol was accumulated at the end of cultivation. Thus, even by applying an automated smooth ramp to *q*_s_ values below *q*_s max_ methanol accumulation could not be prevented. This might be of great interest for bioprocess engineers when it comes to conducting an automated fed-batch process at a larger scale.

#### Comparison of the different dynamic feeding profiles

**Fig. 6:**
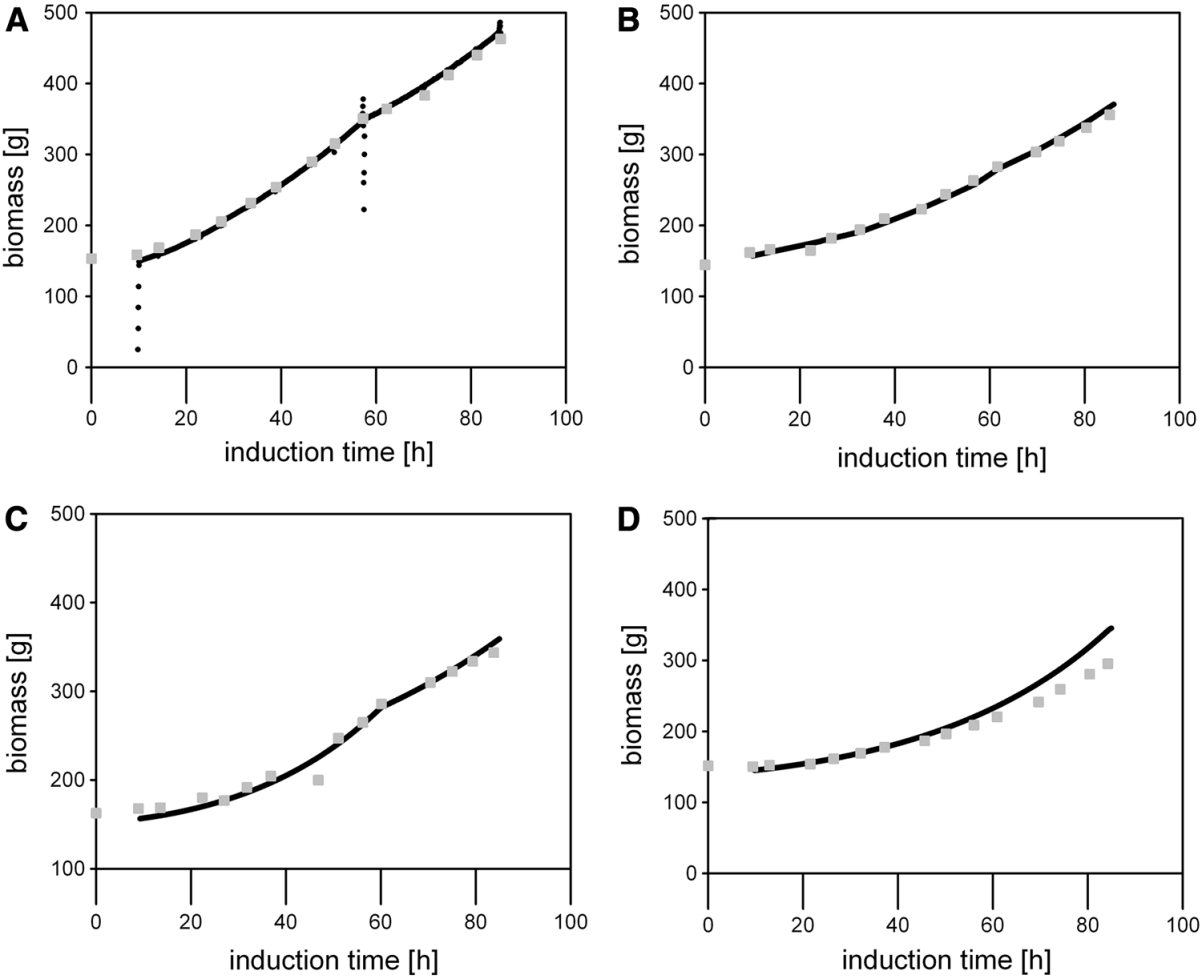
Online calculated biomass concentrations versus offline determined dry cell weight values in all fed batches conducted in this study. **a** FB1; **b** FB2; **c** FB3; **d** FB4. *Black dots*, online calculated biomass concentrations; *dark grey squares*, offline determined dry cell weight values

The total induction time in all fed batches conducted was very similar, allowing a direct comparison of metabolic data and methanol accumulation disregarding any potential time effect [14]. For all the cultivations, similar values and trends for the biomass and carbon dioxide yields were determined: during the first 40 h of induction, *Y*_*X/S*_ stayed almost constant at values close to 0.38 Cmol Cmol^−1^ before a slight decrease of *Y*_*X/S*_ was observed. The $Y_{{{\text{CO}}_{2} /{\text{S}}}}$ in all the cultivations was stable at more than 0.6 Cmol Cmol^−1^ and only increased slightly after 40 h of cultivation which, together with *Y*_*X/S*_ and considering methanol accumulation (actually set at this time point), resulted in C balances close to 1.0. Apparently, cell physiology changed over time causing the decrease in the biomass yield and the concomitant accumulation of methanol. The accumulation was more pronounced when the methanol feed corresponded to *q*_s_ values close to *q*_s max_, underlining the validity of this important strain characteristic parameter. When we compared the online calculated biomass concentrations based on *Y*_*X/S*_ and the online recorded feed to offline determined DCW values, we only observed slight deviations underlining the validity of the online calculator tool (Fig. 6).

As depicted in Fig. 6, the online calculated biomass concentrations and the offline determined DCW showed a very high correlation. Only in FB4, more pronounced deviations of these two values were observed, which was actually set on after around 40 h of induction (Fig. 6d). This is in accordance to the time point when also *q*_s real_ and *q*_s set_ started to depart significantly (Fig. 5b).

In terms of methanol accumulation, experiment FB1, where a methanol feed corresponding to a constant *q*_s_ of 1.75 mmol g^−1^ h^−1^ was automatically provided by an online calculator tool, is the preferred strategy. At the end of cultivation, only 2.0 g L^−1^ methanol accumulated in the cultivation broth. The automated feeding approach describing linear ramps of *q*_s_ (FB3 and FB4) resulted in a more than fourfold higher methanol accumulation of up to 9.0 g L^−1^. The dynamic feeding strategy of manually increasing the *q*_s_ setpoint stepwise with step sizes of 24 h (FB2), which describes a profile where the cells are repeatedly stressed but can also adapt in between, resulted in less methanol accumulation at the end of the cultivation (i.e. 6.5 g L^−1^).

### Productivity

**Fig. 7:**
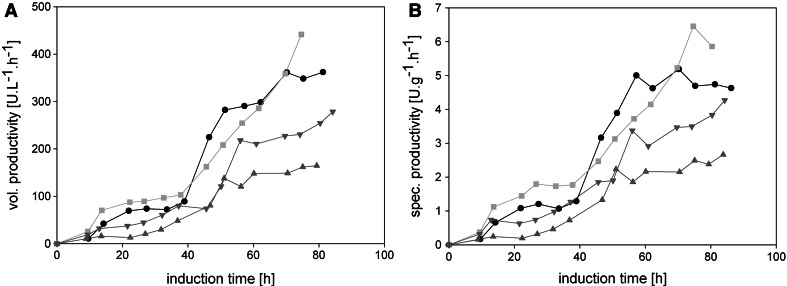
Volumetric and specific productivities. **a** Volumetric productivity (*r*_p_). **b** Specific productivity (*q*_p_). *Black dots*, FB1; *light grey* *squares*, FB2; *dark grey triangles* facing up, FB3; *dark grey triangles* facing down, FB4

In terms of avoiding extensive methanol accumulation, FB1 was determined to be superior to the other strategies tested. However, one key parameter in industrial production processes is the productivity, which is why we determined and compared the volumetric (*r*_p_) and the specific (*q*_p_) productivity in all the cultivations performed (Fig. 7).

The volumetric productivities in FB1 and FB2 were much higher than in FB3 and FB4. The strategy at a high constant *q*_s_ of 1.75 mmol g^−1 ^h^−1^ (FB1) gave a similar volumetric productivity compared to the dynamic stepwise feeding method (FB2) during the first 70 h of induction time—only then we observed that the stepwise increase of the feeding setpoint gave a steeper increase than the more static strategy (Fig. 7a). This phenomenon could be especially important for subsequent long-lasting production processes. The automated feeding profile describing a steep linear ramp of *q*_s_ (FB3) gave the lowest volumetric productivity. The specific productivity showed a similar pattern (Fig. 7b). Although this observation is in accordance to our previous study, the positive effect of the dynamic stepwise strategy was more pronounced compared to a feeding profile at a constant *q*_s_ [14]. We assume that the reason therefore lies in the different ways of manually adjusting the feeding rate during the single *q*_s_ steps: in our previous study, we manually adjusted the feeding setpoint according to the required *q*_s_ setpoint every 8 h [14], whereas in the present study we performed these adjustments every 4 h. Consequently, the cells experienced more pronounced oscillations in the specific substrate uptake rate *q*_s_ in our previous study. Actually, the positive effect of oscillating feeding strategies on the productivity has been shown before (e.g. [18, 19]).

In general, for all feeding strategies in this study, two phases regarding productivity could be distinguished: in the first 40 h of induction, both *r*_p_ and *q*_p_ increased steadily, before a steeper increase in both values until the end of cultivation was observed (Fig. 7). Interestingly, this increase after around 40 h of induction happened independent of the feeding strategy applied.

This is similar to the results we have obtained in one of our previous studies, where we found a time-dependent trajectory of *q*_p_ [14], but interestingly all experiments carried out in our present study showed changes in cell physiology (*Y*_*X/S*_, $Y_{{{\text{CO}}_{2} /{\text{S}}}}$, methanol accumulation) at exact the same time point (Figs. 2, 3, 4, 5). Thus, the apparent change in metabolism and the increase in productivity of *P.* *pastoris* at around 40 h of induction time might cause the observed changes in the biomass yield and the consequent accumulation of methanol. However, this is speculative and remains to be elucidated in detail.

**Fig. 8:**
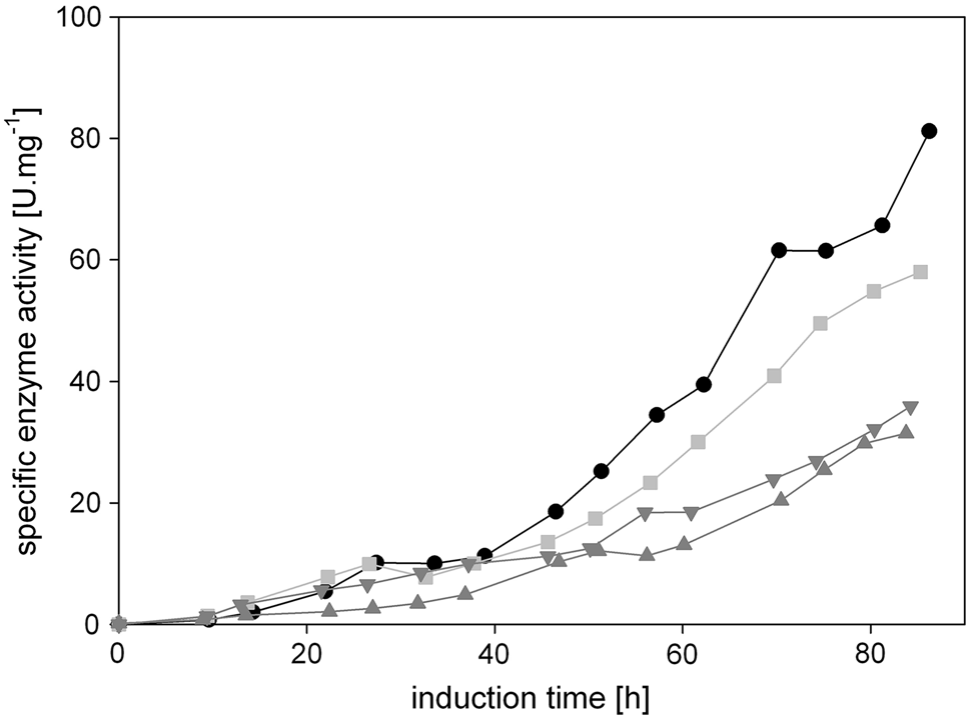
Specific enzyme activity. *Black dots*, FB1; *light grey squares*, FB2; *dark grey triangles* facing up, FB3; *dark grey triangles* facing down, FB4

However, not only the productivity is a key parameter in bioprocess technology, also the quality of the product has to be considered. Thus, we determined and compared the specific enzyme activity (U/mg total protein in the cell-free cultivation broth) during the different fed-batch cultivations (Fig. 8).

In terms of product quality, the feeding strategy at constant high *q*_s_ of 1.75 mmol g^−1^ h^−1^ (FB1) turned out to be superior to the other strategies tested. The amount of active HRP of the total protein in the cultivation broth was more than twofold higher compared to the strategy of linearly increasing the *q*_s_ setpoint (FB3 and FB4, accelerated exponential feeding profile). Although stepwise adjusting the feeding rate to *q*_s max_ (FB2) gave slightly higher productivities at the end of cultivation (Fig. 7), the quality of the product was lower compared to FB1. Apparently, by repeatedly stressing the cells, they secreted more contaminating proteins into the cultivation broth. This hampers a subsequent downstream process and should thus be minimized.

## Conclusions

In this study, we compared different dynamic, physiological feed forward strategies for a recombinant *P.* *pastoris* strain with respect to cell physiology, methanol accumulation, productivity and product quality. Based on our previous findings, we fine-tuned and automated different dynamic feeding strategies and analysed their effects in detail.

Our findings can be summarized as follows:

- A feeding profile based on *q*_s_, where the feeding rate was stepwise increased after adaptation to correspond to a constant automatically controlled *q*_s_ value below *q*_s max_ (FB1), resulted in lowest methanol accumulation at the end of induction.
- The volumetric and specific productivity in fed batches, where the feed was either automatically controlled at a high *q*_s_ setpoint (FB1) or stepwise increased to *q*_s max_ (FB2), were very similar. Only at the end of cultivation, the more dynamic method proved to be superior in terms of productivity.
- The strategy of automatically feeding the cells at constant *q*_s_ below *q*_s max_ (FB1) resulted in the highest product quality.
- After a certain induction time, the productivity rose abruptly accompanied by a decrease in the biomass yield and a consequent accumulation of methanol independent of the feeding strategy applied. However, methanol accumulation was more pronounced when the feed corresponded to *q*_s_ values close to *q*_s max_.
- The implementation of an online calculator tool allows the automation of a dynamic feeding regime based on *q*_s_ provided that the biomass yield of the cells stays constant over time. The only requirement to automatically feed the cells according to Scheme 1 is the knowledge of the biomass yield on the respective substrate and the exact concentration of the feed. When *Y*_*X/S*_ changes over time (e.g. caused by induction events), as observed in the present study, we suggest modifying the online calculator tool to use $Y_{{{\text{CO}}_{2} /{\text{S}}}}$, which can be determined online, and online measured methanol concentration (e.g. by online HPLC or online GC) in the cultivation broth to adjust the feeding rate and to allow a more precise automated feeding.

Summarizing, a dynamically changing stepwise physiological feeding strategy resulted in the highest specific productivity, whereas a dynamic feeding profile at high constant *q*_s_ gave the lowest methanol accumulation and the highest product quality. Since the latter strategy could also be controlled automatically by implementing an online calculator tool, we recommend using this strategy especially for bioprocesses where the biomass yield does not change over time.

## Abbreviations

∆time_adapt_: Time for adaptation of the culture to the new substrate (methanol) (h)
μ: Specific growth rate (h^−1^)
CER: Carbon dioxide evolution rate (mmol L^−1^ h^−1^)
c_s,feed_: Substrate concentration in feed (g/l)
*e*: Euler’s number
*F*, *F*_0_: Feeding rate, initial feeding rate (g h^−1^)
HRP: Horseradish peroxidase
*k*: Exponential coefficient
*m*_feed,in_: Amount of feed added (g)
M_s_: Molecular weight (g mol^−1^)
Mut^S^: Methanol utilization slow phenotype
PID: Proportional-integrative-derivative controller
*q*_p_: Specific productivity of horseradish peroxidase (U g^−1^ h^−1^)
*q*_s_: Specific substrate uptake rate (mmol g^−1^ h^−1^)
q_s max_: Maximum specific substrate uptake rate (mmol g^−1^ h^−1^)
ρ_feed_: Density of feed (g L^−1^)
*r*_p_: Specific productivity of horseradish peroxidase (U L^−1^ h^−1^)
rpm: Rounds per minute
$\dot{S}$: Substrate feed rate (g h^−1^)
*S*_in_: Amount of substrate added (g)
*t*: Time (h)
vvm: Volume gas flow per volume liquid per minute
*X*_*n*_: Total biomass at time point *n* (g)
$Y_{{{\text{CO}}_{2} /{\text{S}}}}$: Yield coefficient of carbon dioxide respective to methanol (Cmol Cmol^−1^)
*Y*_*X/S*_: Yield coefficient of biomass respective to methanol (Cmol Cmol^−1^)
